# Five Advances in the Last 50 Years: Liver Malignancies

**DOI:** 10.1002/wjs.70327

**Published:** 2026-04-11

**Authors:** Alex B. Blair, Timothy M. Pawlik

**Affiliations:** ^1^ Department of Surgery The Ohio State University Wexner Medical Center and James Comprehensive Cancer Center Columbus Ohio USA

## 1 Introduction

Surgical management of liver malignancies has experienced continual and dramatic advancement since the first reported attempts of liver resections in the late 19th century by German surgeon Carl Langenbuch [1]. Improved understanding of liver anatomy, segmental organization and vascular control by pioneering surgeon‐scientists such as James Pringle, Sir James Cantlie, and Claude Couinaud laid the initial groundwork for safer, more effective resection of hepatic tumors [2, 3, 4]. Nonetheless, by the 1970s clinical challenges persisted: effective systemic therapies were scarce and the limits for safe hepatectomy were not clearly defined. Major hepatectomy remained a high‐risk, morbid procedure with 30‐day operative mortality exceeding 25% even among the most experienced centers worldwide [5, 6]. Over the past half‐century, the multidisciplinary treatment landscape for liver malignancies has undergone a profound transformation. Advancements in anesthesia, surgical technique, percutaneous interventions, molecular biology, and immunotherapy have reshaped outcomes for these patients. The past 50 years have ushered in a new era of treatment. Herein we discuss the five most influential medical advancements that have fundamentally altered the management and prognosis of patients with liver malignancies.

## 2 Perioperative and Intraoperative Management

Curative‐intent resection of primary and secondary liver malignancies remains the gold standard when feasible. The earliest attempts of liver surgery were perilous procedures marked by massive blood loss, frequent transfusions and high morbidity. While the potential for bleeding still exists, a deeper understanding and refined command of complex hepatic anatomy has dramatically improved the safety of hepatic resection. A seminal advancement in the perioperative management of patients undergoing liver resection was the introduction of low central venous pressure (CVP) anesthesia. Initial data demonstrated that patients undergoing major hepatectomy with a maintained CVP of 5 mm Hg or less had substantially reduced blood loss and shorter hospital stays [7]. These findings have been consistently reproduced across multiple high‐volume centers worldwide with low CVP leading to lower median intraoperative blood loss, reduced 30‐day and in‐hospital mortality, fewer intensive care unit days, shorter overall length of stay, decreased blood transfusion requirements, improved preservation of postoperative renal function and an almost complete elimination of intraoperative deaths [8, 9, 10]. Contemporary practice increasingly operationalizes low‐CVP principles without routine central venous catheter placement, relying instead on goal‐directed resuscitation using dynamic indices of fluid responsiveness (e.g., pulse pressure/stroke volume variation) and mean arterial pressure to guide intraoperative fluid management.

## 3 Systemic and Immunotherapy for Primary and Secondary Liver Malignancies

Advances in systemic therapy have expanded the pool of patients with primary and secondary liver malignancies eligible for surgical resection. While traditional systemic cytotoxic chemotherapy had response rates of only 10%–20%, the introduction of more potent, triplet, and combination regimens (e.g., FOLFOX, FOLFIRI, FOLFOXIRI) combined with molecularly targeted agents has more than doubled the response rate among patients with colorectal liver metastasis [11, 12, 13]. In turn, more effective systemic chemotherapy has allowed for down‐sizing of initially unresectable liver metastases to enable curative‐intent surgical resection. In addition, the use of immunotherapy for MSI‐high patients with colorectal liver metastasis has become standard first‐line therapy [14]. The randomized SHARP trial was among the first to demonstrate that sorafenib, a multikinase small molecule inhibitor, was cytostatic therapy with a modest survival benefit [15]. More recently, checkpoint inhibitor therapy has increasingly become standard of care for patients with primary liver cancers. The IMBrave50 trial demonstrated that Bevacizumab (anti‐VEGF) and Atezolizumab (anti‐PD‐L1) had a survival advantage and a near 90% response rate compared with just 19% in the sorafenib control arm [16, 17]. The HIMALAYA trial demonstrated that combination immunotherapy regimens with both PD‐L1 and CTLA4 based therapies also improved outcomes compared with first line sorafenib therapy for unresectable HCC [18, 19]. Similarly, the TOPAZ‐1 and KEYNOTE‐966 trials reported that use of immunotherapy was associated with an improved overall survival among patients with advanced ICC compared with cytotoxic chemotherapy alone [20, 21, 22]. Advances in systemic therapy have redefined the treatment landscape for liver malignancies and have ushered in a new era of durable responses and improved survival.

## 4 Liver Augmentation

The criteria for resectability have been expanded to include any patient in whom all disease can be removed with a negative margin and who has adequate hepatic volume/reserve. In essence, instead of resectability being defined by what is removed, decisions concerning resectability now center around what will remain after liver resection [23]. In turn, techniques to increase the volume of the remnant liver are critical to facilitate the possibility of liver resection. Preoperative volumetric assessment with cross‐sectional imaging has allowed for a more quantitative manner to determine the future liver remnant (FLR). Among patients with an inadequate FLR, several techniques have been developed to augment liver volume. Portal vein embolization (PVE) was first described in the late 1980s as a strategy to induce antitumor effects in patients with HCC [24]. Portal vein inflow occlusion was noted, however, to stimulate growth of the FLR, thereby minimizing the risk of postoperative liver dysfunction. Subsequently, PVE has been widely adopted as a safe means to induce hypertrophy of the contralateral liver to allow for safe resection with a decreased risk of postoperative liver insufficiency [24]. In turn, PVE can expand the candidacy of patients for major hepatectomy among patients previously deemed to have insufficient FLR volumes [25]. More recently, preoperative total venous deprivation through the addition of concomitant hepatic vein embolization (HVE) to PVE has been proposed to accelerate FLR hypertrophy. The DRAGON 1 trial demonstrated that combined PVE and HVE was safe, associated with low morbidity and yielded higher rates of subsequent resection in initially upfront unresectable colorectal liver metastases [26]. Associating liver partition and portal vein ligation for staged hepatectomy (ALPPS) leverages this same hypertrophy principle utilizing a surgical technical approach [27, 28].

## 5 Liver Directed Therapies

The blood supply to the liver is distinctive, receiving inflow from both the portal venous system and the hepatic arteries. While normal hepatic parenchyma derives the majority of its perfusion from the portal circulation, primary and metastatic liver tumors preferentially rely on arterial perfusion [29]. This physiologic distinction provided rationale for arterially directed therapies designed to selectively deprive tumors of their blood supply while inducing ischemia and necrosis. In 1983, Konno and colleagues described marked antitumor effects, including both tumor regression and decreased serum alpha‐fetoprotein levels, following selective hepatic arterial administration of an anti‐cancer agent in patients with HCC [30]. Transarterial chemoembolization (TACE) delivers microspheres loaded with antineoplastic drugs into the tumor's arterial blood supply. These particles serve as both a means for therapeutic delivery and an embolic agent to induce ischemia thereby enhancing the local concentration and cytotoxic effects of the chemotherapeutic payload. Over the ensuing decades, extensive investigations have refined the combination of anti‐cancer drugs, embolizing agents and microsphere platforms across a range of tumor types including HCC, ICC, and neuroendocrine liver metastases [31, 32, 33, 34]. Other arterially directed therapeutic strategies have emerged that similarly take advantage of the preferential arterial blood supply of tumors while largely sparing healthy liver parenchyma. For example, Transarterial Radioembolization with Yttrium‐90 beads (TARE‐Y90) delivers radiation‐loaded microspheres allowing maximal high‐dose radiation exposure directly to tumors. In selected patients with HCC, this approach may be preferable for tumor control and survival compared with TACE [35, 36]. A meta‐analysis of 21 studies of patients with unresectable ICC malignancy reported a disease control rate exceeding 80% and median OS of 12.7 months associated with TARE‐Y90 [37]. Hepatic arterial infusion pumps (HAIP) are an additional approach that have leveraged the concept of preferential arterial blood supply with delivery of continuous high‐dose Floxuridine chemotherapy directly to the liver via a catheter in the gastroduodenal artery [38, 39]. While not universally utilized, there is growing application of the HAIP approach to treat colorectal liver metastases and locally advanced ICC [40, 41, 42]. In fact, the PUMP‐2 trial recently reported a 31.5% 3‐year OS for patients with unresectable cholangiocarcinoma started on HAIP chemotherapy [43]. Ongoing clinical trials and investigation of novel combination strategies continue to define the evolving role of arterially directed therapies and lay the foundation for the next‐generation of treatment paradigms for liver malignancies [44, 45].

## 6 Transplant Oncology

From an oncologic standpoint, liver transplantation provides the widest possible surgical margins while also offering the opportunity to restore normal liver function. Liver transplantation has downside risks including immunosuppression and rejection, risk of secondary malignancies, as well as the challenge of limited donor organs. Early evolution of liver transplant for cancer included a need to overcome initial technical challenges, as well as understand optimal immunosuppression. The initial experience with liver transplantation for liver malignancies was characterized by high rates of recurrence and poor long‐term outcomes [46]. During the 1980s, recurrence was as high as 75% and 5‐year survival was only 15%—likely secondary to poor biologic selection of transplant patients with advanced tumor stages, macrovascular invasion, and the presence of extrahepatic disease [47, 48]. These poor outcomes coupled with donor organ wait times and treatment costs led to questioning the viability of transplantation for malignancy with the US Department of Health even declaring HCC a contraindication for transplant in 1989. The landscape of liver transplantation for malignancy was dramatically reshaped following establishment of clearly defined guidelines for patient selection, later deemed the “Milan Criteria”: single HCC tumor 5 cm or less, or a maximum of 3 tumors each with a maximum diameter of 3 cm [49]. Based on these criteria, patients were able to achieve 4‐year survival of 75%, like that of transplant for nonmalignant disease [49]. When analyzing nearly 50,000 HCC liver transplant patients between 1987 and 2001, the implementation of the Milan Criteria led to a marked improvement of survival from 25.3% (1987–1991) to 61.1% (1996–2001) [50]. Improvement in patient selection and systemic therapies have expanded the role of transplant for other oncologic indications. For example, liver transplantation is a well‐accepted and effective surgical strategy for patients with small, early‐stage, unresectable perihilar cholangiocarcinoma who have undergone neoadjuvant chemoradiation and assessment of regional lymph nodes [51, 52, 53]. More recently, liver transplantation has had an increasing role for well‐selected patients with unresectable ICC and CRLM. Encouraging data related to transplantation for patients with unresectable ICC after stability on neoadjuvant therapy has been reported in the prospective TESLA trial, as well as transplantation for unresectable CRLM in the TransMet trial [54, 55, 56]. As these data mature so will our outstanding of patient selection and tumor biology, which will help refine the role of liver transplantation for oncologic indications.

## 7 Conclusion

Over the past 5 decades, the multidisciplinary management of liver malignancies has undergone transformative advances resulting in improved outcomes and longer patient life. The adoption of low CVP anesthesia addressed the initial longstanding challenges of intraoperative hemorrhage enabling safer, more meticulous surgery. Advances in chemo‐ and immune‐therapy have reshaped the cancer care paradigm through more effective treatment of advanced disease with increased ability to convert disease to resectability. PVE and liver augmentation has facilitated the ability to perform more extensive hepatic resection procedures while reducing postoperative liver insufficiency. Arterially directed therapies (TACE, TARE‐Y90, HAIP) provide effective locoregional control by exploiting tumor arterial perfusion. Liver transplantation—with removal of the entire liver to extirpate all hepatic disease—continues to emerge as a tenable option for select patients with unresectable disease.

While these five milestones warrant being highlighted, there are many other innovations that have moved the field forward over the last 50 years. Some of these advances include Hepatitis C therapy, improved screening and imaging with contrast‐enhanced MRI and development of a standardized reporting system (i.e., Li‐RADS), as well as thermal and high frequency ultrasound ablation. The next 50 years will undoubtedly witness additional advances including xenotransplantation, expansion of precision medicine, as well as improved biomarkers (i.e., liquid biopsy/circulating tumor cells). Regardless of the specific innovation, the future offers significant promise to broaden treatment eligibility, enhance tumor control, and further extend the survival of patients with primary and secondary liver malignancies.

## Author Contributions

**Alex B. Blair:** conceptualization, writing – original draft, writing – review and editing. **Timothy M. Pawlik:** conceptualization, writing – original draft, writing – review and editing.

## Funding

The authors have nothing to report.

## Conflicts of Interest

The authors declare no conflicts of interest.

## Data Availability

Data sharing not applicable to this article as no datasets were generated or analyzed during the current study.
